# Balancing fertility and livelihood diversity in mixed economies

**DOI:** 10.1371/journal.pone.0253535

**Published:** 2021-06-24

**Authors:** Joseph V. Hackman, Karen L. Kramer

**Affiliations:** Department of Anthropology, University of Utah, Salt Lake City, Utah, United States of America; University of California Santa Barbara, UNITED STATES

## Abstract

Mixed economies provide a unique context for testing theories of fertility change. Because they have a stake in two traditions, mixed-economy households balance the demands of both a labor-based subsistence economy, which benefits from a large family, and a wage-labor economy, which benefits from reduced fertility. Additionally, household size changes over the course of its life-cycle and shapes available economic opportunities. Here we argue that in mixed economies, fertility may reflect opportunities for livelihood diversity rather than simply responding to the restricted socioeconomic benefits of small families. While low fertility may in some cases have an economic benefit, low fertility can also limit the livelihood diversity of a household which is a key strategy for long-term economic success. We test this prediction with longitudinal data from a Maya community undergoing both a sustained decline in fertility and rapid integration into the market economy. Using household-level fertility, number of adults, and livelihood diversity at two time points, we find that household size is positively related to livelihood diversity, which in turn is positively related to household income per-capita. However, household size also has a negative association with income per capita. The results reflect a balancing act whereby households attempt to maximize the economic diversity with as few members as possible. Broadly, these results suggest that theories of fertility decline must account for how households pool resources and diversify economic activities in the face of increasing market integration, treating fertility as both an outcome and an input into economic and reproductive decision-making.

## Introduction

How households transform over the course of economic and social transitions has long been a central question of social sciences. Under its various monikers of modernization, industrialization, urbanization, and market integration, studies of the cascading effects of market involvement on indigenous and small-scale communities have had consequences on resource use [1], health and well-being outcomes [2, 3], changes in ecological knowledge [4, 5], and prosocial behaviors [6, 7]. Over the last 60 years particular attention has been paid to the decline in fertility and the rise of economic inequality as two robust patterns emerging from market development [8–10].

In the case of fertility, motivated by an evolutionary puzzle [11, 12] where increasing access to resources results in a decline of reproductive output, a large body of work has investigated how market opportunities change payoffs to reproduction and parental investment [13–16]. Economic or investment models of fertility transitions argue that fertility declines are associated with changes in the prevailing economic system, primarily the transition from subsistence production to competitive wage-labor [9], where market opportunities increase the costs associated with large families [13, 17]. In market economies, the payoffs to investments in offspring do not diminish until very high levels, leading to an increase in the costs of rearing offspring, and consequently an overall reduction in total fertility. In these contexts, and contrary to optimization assumptions, the trade-offs between total offspring and investment-per-offspring do not appear to maximize fitness in the long-run [18–20]. While low fertility never appears to have a fitness payoff, in market economies it does seem to have an economic dividend. Indeed one feature of most fertility transitions is a negative association between wealth and fertility [21–23], whereby low fertility is associated with greater levels of wealth. While this negative relationship was once deemed an intractable problem for human evolutionary sciences, recent re-evaluations have found rather a weakened, variable, and clustered relationship between wealth and fertility [9, 24–27].

A tacit assumption in the analyses of how fertility changes with modernization, is that the economic transition follows a linear progression from traditional, subsistence production, to a wage-labor and market-integrated economy. However, in many small-scale and subsistence populations the process of market integration is not a straightforward replacement of one economy with another [5]. Rather, the process creates *mixed economies*, where households combine subsistence production and wage-labor. In mixed economies, households not only engage in both the market economy and subsistence production, they also maintain both traditional sharing and cooperative relationships [28, 29] as well as pursue new institutional and other social relationships. Furthermore, mixed economies may be stable and persistent rather than a temporary stage in the transition to the market economy [30]. This context creates unique trade-offs that households must navigate as they balance traditional economic and social behaviors with novel and often uncertain market opportunities.

While mixed economies are often viewed as transitional, maintaining both subsistence production and traditional cooperative relationships while folding in new economic and social opportunities may serve to enhance livelihood stability [31]. Indeed, for at least 40 years, scholars have characterized rural livelihood strategies in contemporary lower and middle income countries as maintaining and continuously adopting a diverse portfolio of economic activities for long-term economic stability [32–34]. Across rural contexts, households with diversified economic activities are consistently less vulnerable and more resilient to economic and climate related shocks [31, 35–39]. The stability and persistence of mixed economies suggests that households engage in a demographic balancing act by having a stake in two worlds. Thus, understanding fertility transitions involves identifying how households navigate the trade-offs associated with the economic diversification that characterizes mixed economies, particularly when traditional production relies on kin-based labor pools, putting a premium on larger families.

Economic theories of fertility decline often emphasize quantity-quality trade-offs where market opportunities impose costs on excess production of children and place premiums on investments in education. Broadly, the benefits to increased formal schooling are well-known. Market economies increase the importance of skills acquired through formal education and training [40, 41]. Among other things, education is positively associated with income, and is a primary means for social and economic mobility in market economies [42, 43]. However, school fees, supplies, uniforms, and transportation carry direct costs that can present heavy economic burdens for parents and households [44, 45], as well as indirect opportunity costs in limiting the ability of children to contribute to the household production, and provide allocare [46, 47]. Additionally, when choosing to invest in education, the long-term returns to schooling can be uncertain and variable, depending on access and distribution of opportunities to use education for social and economic mobility [48–50].

Demographic processes also directly shape household composition, which determines the ways in which households can navigate economic production. Often in traditional societies, household consumption and production vary with both size and the age-sex composition of the household [51]. Among other things, migration, post-marital residence, and marriage patterns all contribute to determining the structure and temporal composition of a household. One key demographic shift associated with household composition is the decline in fertility associated with increasing access to market economies. In the contexts of mixed economies, a commitment to low-fertility may either 1) be fraught with uncertainty in terms of the availability of market opportunities [52] and 2) may limit a household’s ability to practice customary forms of economic production.

Mixed economies provide a unique opportunity to uncover the links between declining fertility and shifting patterns of inequality. Here we suggest that rather than an arms race to low fertility, in mixed economies fertility reflects the loosened, variable, and often uncertain economic constraints on rearing offspring. While a low-fertility high-investment strategy may indeed increase competitiveness in the wage-labor economy, poor access to good jobs and the persistence of alternative economic activities provide flexibility in how households meet labor needs and maximize livelihood diversity.

This contrasts with standard evolutionary and economic predictions, which argue that low fertility will reap rewards associated with market opportunities. Rather than just a linear negative association between fertility and household income, we expect income will increase with the economic diversity of a household, which will require additional adults to capitalize on. The balancing of these opposing forces–smaller households being able to increase income per capita, but larger household being able to increase economic diversity, and thereby income per capita—generates a reproductive balancing act. In other words, reducing family size too much might constrain the ability of a household to diversify economically despite potential income benefits, while too high fertility may limit the ability to invest sufficiently in children to take advantage of new opportunities. Additionally, limits to the total number of potential economic activities means increasing household sizes beyond a point may create diminishing returns for adding additional adults. We test this prediction with longitudinal data from a Maya community undergoing both a sustained decline in fertility alongside rapid integration into the global market economy.

### Predictions

**Fig 1:**
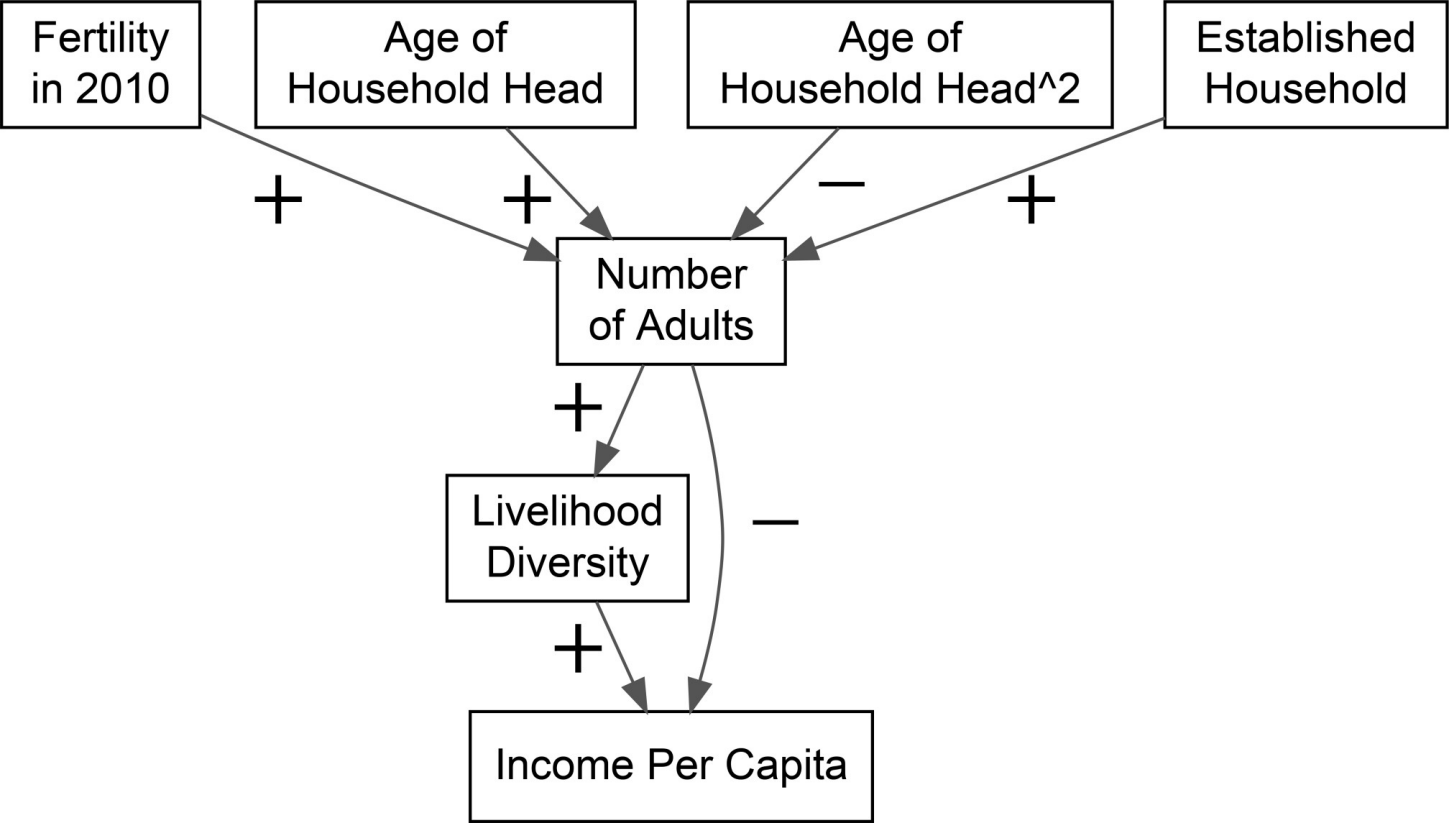
Schematic model depicting the predicted relationships between number of adults, livelihood diversity, and income per capita. Livelihood diversity increases with increasing number of productive adults. Livelihood diversity also has a positive impact on resource flows through the household (income per capita). However, household size will have a direct negative impact on income per capita.

Here we argue that in mixed economies, fertility responds to building diversified livelihoods, rather than solely to the opportunity costs associated with high parental investments in children and payoffs to pursue pure wage-labor (Fig 1). We test four predictions regarding fertility behaviors and household economic activities in mixed economies. In particular we examine how fertility decisions shape the number of (productive) adults which in turn influence household livelihoods and resource flows (income) in the future. Here, productive adults refer to all household members between the ages of 15 and 70. Using longitudinal data across a 7-year time period, we first expect that past fertility decisions will condition current number of adults (P1). Specifically, we expect households with higher fertility of all adults in 2010 will have more working adults in 2017. Second, number of adults will be positively associated with livelihood diversity (P2). Households with more adult members are better able to diversify economic activities. Third, livelihood diversity will be positively associated with household economic outcomes, measured here as net resource flows or income per capita (P3). Finally, after accounting for livelihood diversity, number of adults will be negatively associated with lower income per capita (P4). Given the limited types of economic opportunities, households may face decreasing marginal returns for adults engaged in similar economic activities.

### Ethnographic context

Among the Yucatec Maya households consist of a nuclear or multigenerational family and are the unit of consumption and production, across which labor, food and other resources are pooled [51, 53]. The Maya readily identify these cooperative groups, describing in their words, “those who live together, eat together and work together”. Household composition fluctuates as children mature, marry and have children of their own. At marriage, young women often, but not always, move into their husband’s household. The young couple builds a separate sleep structure, but is otherwise economically embedded in the established household. When the couple has cleared sufficient land to support themselves, and has the resources to build their own house, a process that may take 5–10 years, they typically form their own household and identify themselves as economically independent. When there are several adult sons, they may remain attached to their natal household until they become the senior members themselves. Many exceptions exist, notably where husbands move into their wife’s household. Marriages most commonly occur among community members (92.3%); endogamy is the exception and similar for males (9.7%) and females (10.4%).

In the early 1990s, all residents (n = 55 households, 316 individuals) made a living as small-scale agriculturists, the household was the unit of production, and each family grew and hunted for its food. Because of the lack of roads and vehicles, transportation to market towns was limited and there was little means to engage in the regional economy, surplus crop production, schooling or wage-labor. Household-level labor pooling and intergenerational resources flows were essential for family survival. In particular, households depended on older children whose contributions to domestic and field work subsidized the costs of younger siblings [53–55]. Given this household organization and the relationship between wealth, labor and family size traditional labor allocation decisions among subsistence farmers in Mexico have largely been modelled as the result of household decision-making processes as opposed to individual ones [56].

The ejido land tenure system, instituted following the Mexico Revolution prescribed collective ownership of agricultural land, guaranteeing all community members relatively equal and adequate access to the means of production, firewood and other resources. But is also limited the ability to accumulate land, creating low levels of inequality. The Gini coefficient for land distribution was 0.14, which is significantly lower than those estimated for material wealth in other agricultural populations (Gini = 0.48) [57]. The community was also characterized by low-levels of livelihood diversification as primary production centered on agricultural subsistence. While a majority of households did engage in some type of wage labor (75%), employment was almost exclusively paid farm work [51].

In the early 2000s, the development of a paved road linking the community to market towns accelerated market integration. Greater mobility changed agricultural production by providing access to tractors and mechanized farming, fertilizers and pesticides and a means to transport crops to market, which opened channels for selling surplus production. Agricultural intensification, and relaxation of the ejido land-tenure system has led to a significant increase in land inequality. The Gini coefficient for land distribution has increased to 0.46, closer to estimates for other agricultural populations [52]. The road also opened up access to education and wage labor as an alternative to agricultural production. These changes directly shape children’s economic value. Schooling has substituted, almost hour for hour, the time previously spent in productive tasks, creating opportunity costs for children to participate in economic activities.

### Changing fertility

Since the opening of the road, completed fertility has been in decline. Average completed fertility (number of living children) in the early 1990s was 6.8 (SD = 2.5). Completed fertility remained high for another decade. However, for women whose reproductive lives were mostly spent after the road opened, completed fertility has declined significantly to under 4 (SD = 3.0) children [52].

A key change underpinning the sustained decline in fertility was the adoption of parity-specific forms of contraception. With access to transportation, more women began giving birth in medical facilities, where they were presented with the option of tubal ligation, typically performed directly after childbirth, and the predominant form of family planning. Tubal ligations were initially adopted by older women at high parities was not associated with smaller completed fertility for almost decade. Younger women, however, increasingly use family planning to limit fertility at lower parities [52].

### Household composition and livelihood diversity

In the context of declining fertility, we might expect that low fertility households are better positioned to invest in emerging education opportunities and wage-labor prospects. However, uncertainty in novel market payoffs changes the benefits to low fertility, as income advantages to additional schooling are often unknown. By contrast, high-fertility, and by extension large households, can come with a cost for high offspring investment strategies. High fertility increases the number of children needed to be schooled, and may produce overcrowded households, which is a well-known indicator of multi-dimensional inequality in Mexico, and is associated with low asset wealth and poor household conditions [58, 59].

We propose that households engage in bet hedging and livelihood diversification in these contexts of uncertain payoffs associated with both intensifying agricultural production and committing to wage labor jobs. Bet hedging and livelihood diversification strategies involve spreading risks across multiple domains, such that households may invest in education and wage-labor opportunities, while simultaneously maintaining agricultural production for both the market and subsistence consumption.

Qualitative studies of subjective poverty show that livelihood diversity is a perceived indicator of household well-being. Studies in larger, more urbanized Maya communities in the Yucatan found Yucatec Maya rank wealthier households as having larger agricultural holdings alongside fixed employment and non-agricultural business opportunities, whereas poorer households had little access to agricultural land, relied on subsistence production or seasonal agricultural wage labor [60]. Furthermore, maintaining a subsistence agricultural base acts as insurance against market fluctuations in both commercial agricultural and wage-labor opportunities [31].

## Materials and methods

### Data collection

To test the four predictions, we use detailed censuses and reproductive histories collected in 2010 and 2017, alongside detailed household economic data collected in 2017. The 2010 Maya research was approved by Harvard University Committee on the Use of Human Subjects in Research #F18643-101, and the 2017 research by University of Utah Institutional Review Board #00093510. Verbal informed consent was obtained from all subjects. Written consent is inappropriate in this cultural context. Many have never signed a document, and we do not wish participants to become accustomed to signing documents that they themselves cannot read. Project aims and protocols are explained in a consent script, first to community comisarios and the local health provider, and then to subjects. This longitudinal panel-design permits testing assumptions about how fertility decisions at one time point influence household composition and economic behavior years later.

### Measures

Four primary variables are used to test our predictions (Table 1). First, to estimate household fertility decisions, we calculate *total fertility* which is the average children ever born of all women in the household age 15 or older in 2010. While this measure collapses fertility decisions across multiple women within a household, sensitivity analyses for alternative methods of aggregating household fertility using only head of household fertility (S1 Table in S2 File) and completed fertility of woman age 40 or older in 2010 (S2 Table in S2 File) gave qualitatively similar results. Second, we calculated *total household size* for 2010 and 2017 by summing the total number of individuals aged 15 years or older living in the household. By 15 adolescents have reached the age of net production, meaning they spend as many hours per day working as adults [51, 53]. The total number of productive adults best measures a household’s potential labor force.

**Table 1:** Mean and standard deviation of model variables.

	Established	New	Overall	P-Value
	(N = 59)	(N = 29)	(N = 88)	
Log Income Per Capita*	9.39 (1.5)	9.65 (0.9)	9.47 (1.3)	0.5
Livelihood Diversity	3.47 (1.1)	3.34 (0.9)	3.43 (1.0)	0.32
Total Fertility in 2010	4.67 (1.9)	4.51 (1.6)	4.62 (1.8)	0.8
Number of Adults in 2017	4.81 (2.1)	3.42 (2.4)	4.34 (2.3)	0.01
Number of Adults in 2010	4.46 (2.4)	7.05 (2.6)	5.28 (2.7)	< .001
Age of Household Head	39.53 (10.0)	29.64 (9.5)	36.16 (10.9)	< .001

* Because income per cap included debt (negative values), the variable was scaled to have a minimum of 1 before log transformation.

Third, *livelihood diversity* is defined as the breadth of engagement in distinct types of livelihood activities [28]. For the year 2017, economic behavior was coded for all adult members of the household over the age of 15. The types of activities include agricultural work, wage-labor, piece work, domestic work, and attending schools. Piece work is best described as craft production for sale in local markets. Furthermore, we include attending school as this measure is intended to capture how households diversify adult time allocation. Livelihood diversity ranges from 1–5 and is the sum of the total number of distinct types of adult economic activities in the household.

To assess household economic status, our fourth variable is net annual household *income per capita*. To calculate net income per capita, we took the sum of all household income from wages, agricultural sales, and any government subsidies, minus any agricultural expenses or other large household expenses. Over the last decade, increasing reliance on commercial fertilizers and pesticides have pushed farmers to rely on credit, escalating yearly debt. In 2018 agricultural debt for farmers averaged 21,818 pesos, the equivalent of $1154 US dollars based on the 2017 conversion rate. Net household income ranged from -53,260 ($2,816 US) to a positive 448,101 pesos ($24,091 US). Since income is aggregated at the household level, we divided total household income by number of adults to estimate income per capita (ranging from -7365.0 to 77,037.5 pesos). Because net income included negative values, we centered per capita income to have a minimum of 1 before log transformation.

Finally, we control for two key traits that capture the position of the household in its lifecycle. First, established household are distinguished from new households. *Established households* were enumerated in 2010 and had the same head of household and largely the same member composition in 2017. *New households* were identified when the adult household heads in 2017 were living in a different household in 2010. Of the 88 households in 2017, 59 (67%) were classified as established households, and or whereas 29 (33%) were newly formed households. We make this distinction because past fertility (average in 2010) is expected to have less of an impact on the number of adults of newly formed households by 2017 than established households. Additionally, among new households, greater livelihood diversity is expected to be associated with higher incomes and total number of productive adults to be negatively associated with income per capita. Lastly, we control for household age using the average age of the male and female head-of-household. As a sensitivity analysis, we also assess the association between the age of just the female head-of-household and fertility. To account for nonlinearities, we include age of household squared in all models.

### Methods and analysis

The primary predictions are tested using a structural equation model to account for the simultaneous effects of fertility on household size and of household size on livelihood diversity and income per capita (Fig 1). The primary model includes all households across both time points, and includes a binary predictor for whether the household was established or new. To examine how the effects of fertility and livelihood diversity vary, the analyses are then stratified by established and new households. Finally, model fit is assessed using a standard Chi square test and the Root Mean Square Error of Approximation (RMSEA) with a threshold of <0.05. RMSEA reflects the divergence of the hypothesized model from the “perfect” model, where a threshold of p<0.05 is interpreted as a good fit [61]. As a sensitivity analysis, we also include in the supplemental materials a model (S3 Table in S2 File) that accounts for the effect of household sex composition on livelihood diversity.

## Results

### Descriptives

The descriptive statistics showed that on average, households engaged in 3.4 different types of livelihood activities and had an annual net income per capita of 12,267 pesos (~$695 US) (Table 1). Total fertility of reproductive age women in 2010 was 4.6, with no differences between established and new households. Stratifying the summary statistics by established and new households revealed key differences between the two. First, new households tended to come from significantly larger households in 2010. For new households, size in 2010 was 7.05 members, while established households were 4.46 [p<0.001]. Additionally in 2017, the size of new households was significantly smaller than established households [p<0.01]. This highlights that new households in 2017 were split from larger households in 2010. Finally, new households were significantly younger than established households with the mean age of heads of 29.64 while established households were a decade older 39.53 [p<0.001].

### Bivariate associations

**Fig 2:**
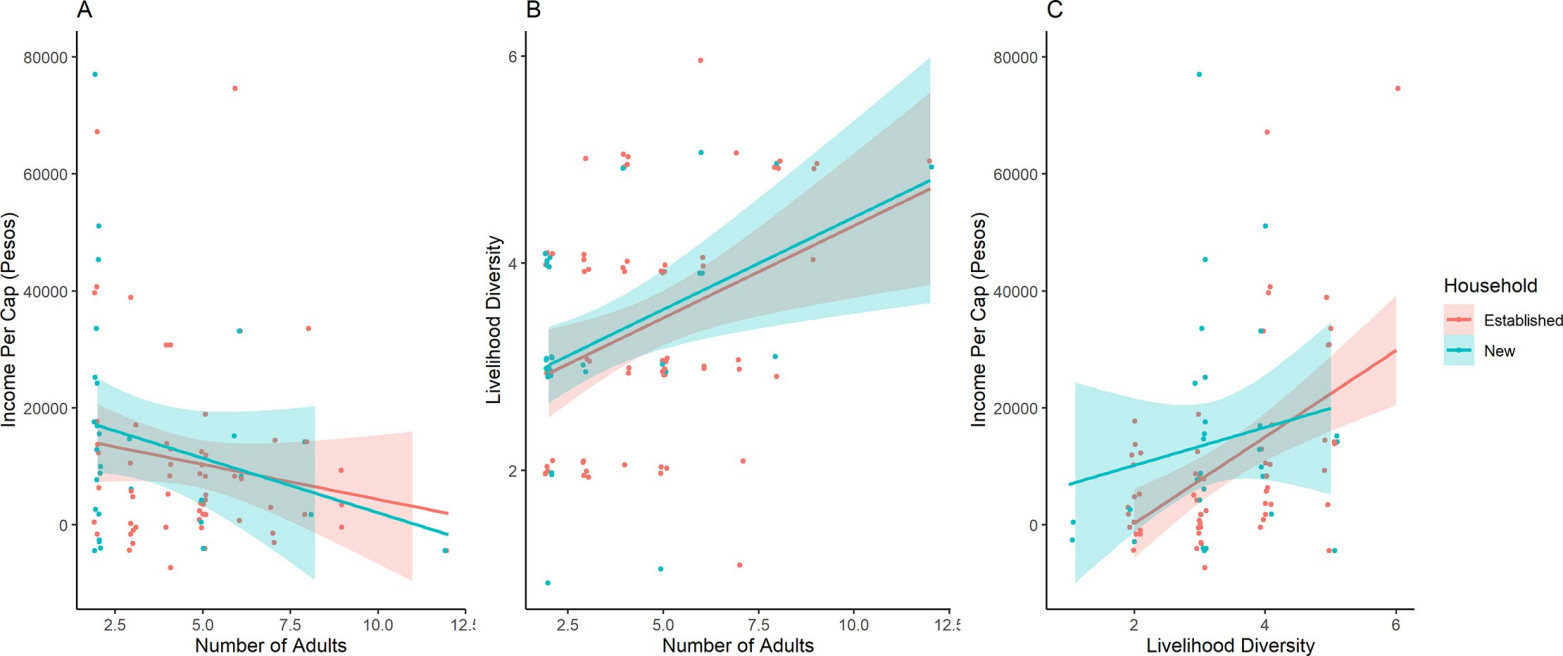
Bivariate associations between income, household size, and livelihood diversity. Association between (A) household size and income per capita, (B) household size and livelihood diversity, and (C) livelihood diversity and income per capita. Data are stratified by established and new households. Shaded areas represent the 95% CI of the OLS line.

Bivariate associations (Table 2, Fig 2) in the full sample show that livelihood diversity is positively associated with net income per capita [r = 0.26; p<0.05]. Additionally, household size is positively associated with livelihood diversity [r = 0.37. p<0.001], indicating larger households have more diverse economic activities. Consistent with the predictions, household size has a negative association with income per-capita, though the correlation was not significant [r = -0.13; p = .20]. The link between fertility in 2010 and household size in 2017 is positive and significant for the full sample [r = 0.23; p = 0.02].

**Table 2:** Correlations between model variables.

Full Sample	Log Income Per Capita	Livelihood Diversity	Household Size	Completed Fertility in 2010	Mean Age of Household Head	Age of Female Household Head
Log Income Per Capita	1.00 (0.00)					
Livelihood Diversity	**0.26 (0.01)**	1.00 (0.00)				
Household Size	-0.13 (0.20)	**0.37 (0.00)**	1.00 (0.00)			
Completed Fertility in 2010	0.03 (0.79)	-0.07 (0.50)	**0.23 (0.02)**	1.00 (0.00)		
Mean Age of Household Head	-0.15 (0.18)	-0.05 (0.62)	**0.41 (0.00)**	0.18 (0.10)	1.00 (0.00)	
Age of Female Household Head	-0.16 (0.15)	-0.05 (0.65)	**0.42 (0.00)**	**0.22 (0.04)**	**0.96 (0.00)**	1.00 (0.00)
Established Households						
Log Income Per Capita	1.00 (0.00)					
Livelihood Diversity	**0.29 (0.03)**	1.00 (0.00)				
Household Size	-0.07 (0.62)	**0.34 (0.01)**	1.00 (0.00)			
Completed Fertility in 2010	0.03 (0.84)	-0.15 (0.25)	**0.31 (0.01)**	1.00 (0.00)		
Mean Age of Household Head	-0.07 (0.59)	-0.18 (0.17)	0.12 (0.35)	**0.26 (0.05)**	1.00 (0.00)	
Age of Female Household Head	-0.10 (0.46)	-0.17 (0.18)	0.16 (0.22)	**0.36 (0.00)**	**0.93 (0.00)**	1.00 (0.00)
New Households						
Log Income Per Capita	1.00 (0.00)					
Livelihood Diversity	0.24 (0.19)	1.00 (0.00)				
Number of Adults	-0.27 (0.14)	**0.44 (0.01)**	1.00 (0.00)			
Completed Fertility in 2010	0.06 (0.76)	0.15 (0.41)	0.07 (0.68)	1.00 (0.00)		
Mean Age of Household Head	-0.18 (0.41)	-0.34 (0.10)	0.39 (0.06)	-0.20 (0.36)	1.00 (0.00)	
Age of Female Household Head	-0.17 (0.45)	-0.28 (0.19)	**0.49 (0.02)**	-0.30 (0.18)	**0.98 (0.00)**	1.00 (0.00)

When stratified by established and new households, bivariate associations show different patterns. For established households, income per capita is positively associated with livelihood diversity [r = 0.29, p<0.05], and livelihood diversity is positively related to number of adults [r = -0.34, p<0.01]. By contrast, for new households, livelihood diversity [r = 0.24, p = 0.19] and number of adults [r = -0.27, p = 0.14] showed no significant association with income per capita, though the size and sign of the associations are suggestive. Finally, for established households, household age was positively associated with fertility in 2010 [r = 0.26, p<0.05] while age showed no association with fertility in new households [r = -0.20, p = 0.26]. Furthermore, when household age was restricted to just women, age showed positive association with completed fertility for established households [r = 0.36, p<0.001] but showed no association within new households [r = -0.3, p = 0.18].

### The full structural equation model

The results of the full SEM show the contrasting effects of livelihood diversity and number of adults on income per capita (Table 3, Fig 2). Consistent with predictions, livelihood diversity shows a positive association with income per capita [B = 0.46, p<0.001], while number of adults shows a significant negative association with income per capita [B = -0.17, p<0.01]. In addition to the negative direct effect of number of adults on income per capita, number of adults had a positive association with livelihood diversity [B = 0.17, p<0.001]. Furthermore, average fertility of reproductive-aged women in the household in 2010 showed a positive, significant association with number of adults in 2017 [B = 0.17, p<0.01]. Age of household head showed effects consistent with established nonlinearities of the household life cycle. Age of household head is initially positively associated with household size [B = 0.42, p<0.001], while age of household head squared showed a negative association [B = -0.0001, p<0.001]. Finally, the model fit indices show the model fit the data well. The Chi-square test indicates good model fit [X = 9.13, df = 6, p = 0.33], as well as the RMSEA [RMSEA<0.04, p = 0.49].

**Table 3:** Structural equation model results.

	Full Model (N = 88)			Established Households (N = 59)			New Households (N = 29)		
Coefficients	B	SE	P	B	SE	P	B	SE	P
**Income**									
Intercept	8.64	0.46	0.00	2.58	0.32	0.00	2.75	0.25	0.00
Livelihood Diversity	0.46	0.14	0.00	0.48	0.18	0.01	0.38	0.19	0.04
Number of adults in 2017	-0.17	0.06	0.01	-0.15	0.09	0.11	-0.17	0.07	0.01
**Livelihood Diversity**									
Intercept	2.67	0.21	0.00	1.33	4.04	0.74	2.75	7.00	0.13
Number of adults in 2017	0.17	0.04	0.00	0.18	0.06	0.00	0.16	0.06	0.01
**Household Size in 2017**									
Intercept	3.81	2.70	0.16	1.33	0.00	0.00	10.56	0.00	0.00
Total Fertility in 2010	0.29	0.13	0.02	0.38	0.13	0.00	-0.02	0.30	0.95
Age of HH	-0.05	0.13	0.73	0.14	0.17	0.43	-0.36	0.43	0.41
Age of HH^2^	-0.0001	0.0001	0.81	-0.002	0.002	0.22	0.00	0.01	0.57
Established HH	2.04	0.60	0.00						
Model Fit Indices	Coef	P		Coef	P		Coef	P	
Chi Square	9.13	0.33		9.71	0.14		7.01	0.32	
	[df = 8]			[df = 6]			[df = 6]		
RMSEA	0.04	0.49		0.10	0.21		0.08	0.37	
	[90% CI = 0.00–0.14]			[90% CI = 0.00–0.22]			[90% CI = 0.00–0.26]		
R-squared									
Income per capita	0.13			0.12			0.21		
Livelihood Diversity	0.15			0.13			0.20		
Household Size	0.20			0.22			0.11		

### The stratified SEM analysis

**Fig 3:**
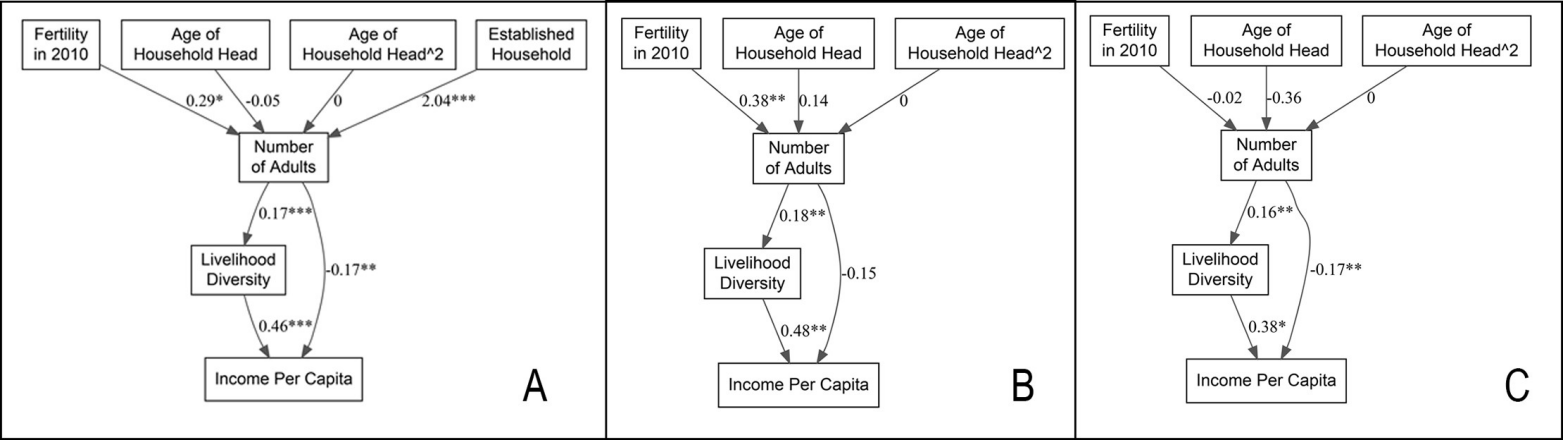
SEM analysis results for the full model (Panel A), and the models for established (Panel B) and new (Panel C) households.

The stratified SEM shows how fertility and livelihood diversity shapes income per capita over the course of the household life cycle (Fig 3). We chose to keep the same model specifications as the full model in order to facilitate comparisons across new and established households, as well as to refine our interpretation of the full SEM model. For the stratified analyses, the model for established households showed poorer, but adequate fit across indices [X = 9.71, df = 6, p = 0.14; RMSEA<0.10, p = 0.21]. However, model fit was better for newly established households, still within thresholds for a close fit [X = 7.01, df = 6, p = 0.32; RMSEA<0.08, p = 0.37].

For established households, results are qualitatively similar to the full mode. Livelihood diversity is positively associated with income per capita [B = 0.48, p<0.01], while number of adults is negatively associated with income per capita [B = -0.15, p = 0.11], however the coefficient falls just outside of statistical significance. As in the full model, number of adults is positively associated with livelihood diversity [B = 0.18, p<0.001]. Additionally, the effects of fertility in 2010 shows a positive association with number of adults in 2017 [B = 0.38, p<0.001].

For new households, results are also largely consistent with the full model. First, livelihood diversity shows a positive association with income per capita [B = 0.38, p<0.05] and number of adults shows a negative association with income per capita [B = -0.17, p<0.01]. These results contrast with those of the bivariate analyses, where these relationships were of similar sign and magnitude, yet failed to reach statistical significance. In the full model, accounting for all variables simultaneously provides a better assessment of the combined effect of number of adults and livelihood diversity on income per capita. Additionally, number of adults maintains a positive association with livelihood diversity [B = 0.16, p<0.01] indicating that the more adults living in newly established households, the more economic activities these households are engaged in. For new households, however, the total fertility of women in 2010 was not a significant predictor of the size of the newly formed household in 2017 [B = -0.02, p = 0.95]. Sensitivity analyses using completed fertility and fertility of just the female head of household are qualitatively similar to the main models presented here (S1 and S2 Tables in S2 File).

In summary, the results indicate that in 2017, households with greater livelihood diversity tend to have more income per capita, with larger households having greater livelihood diversity. However, after controlling for livelihood diversity, larger households tend to have lower income per capita. Balancing economic production is shaped largely by fertility decisions that took place in 2010, where higher fertility in 2010 lead to larger households in 2017. Finally, the demographic effects of fertility decisions in 2010 were most pronounced for established households, while the negative effects of number of adults on income per capita were most prominent for newly formed households.

## Discussion

Mixed economies present an important context for testing theories that emphasize household-level economic production as a key factor shaping fertility decision-making [62]. The combination of traditional production and novel market opportunities means that reproductive decision-making is nested in a broader question of how much households engage in market opportunities. This broader context has clear implications for theories of fertility decline. Generally, economic theories of fertility decline treat populations as belonging to one of two discrete states–those with market opportunities and those without. However, in mixed economies, fertility decisions depend on how households pool resources and take advantage of both traditional production and market opportunities for economic activities.

Our results are consistent with the proposal that mixed economy households face opposing economic forces in determining household income. On one hand, smaller households tended to have greater overall income per capita, consistent with traditional economic theories of fertility transitions. However, households increase income by cultivating a diverse suite of economic activities, and larger households are better able to diversify their livelihood portfolios. These two findings show households face a demographic balancing act in mixed economies. Too large of a household may dilute available income, while too few adults may mean that households need to commit to a single economic strategy.

These results are also consistent with other work on small-scale farmers in rural Mexico, where household capacity to both intensify agricultural production, engage directly with the agricultural economy, and take advantage of emerging wage-labor employment opportunities is a key bet-hedging strategy for managing variability economic production [31]. More generally, livelihood diversity is broadly recognized as a key survival strategy for rural households around the world [35], yet few studies have modelled the demographic necessities associated with such diversification. We extend these findings to suggest that bet-hedging economic strategies influence reproductive decisions in ways not captured by standard economic theories of fertility.

### Implications of the stability of mixed economics for understanding fertility declines

Rural, subsistence, and often indigenous populations commonly maintain a mix of market-based and traditional economic production. Considering these mixed economies as stable strategies may help explain stalled fertility declines that have been documented in Asia, Africa, and Latin America [63–65]. In contexts characterized by unequal, inconsistent, or sparse access to market opportunities, the benefits of reduced fertility may not outweigh the need to diversify livelihoods. Understanding the commitment to and distribution of mixed economic practices is crucial to understand fertility variance as populations experience demographic shifts to low fertility. Economic theories that emphasis the quantity-quality trade-off often focus on the level of the individual or couple. However, as households balance livelihood diversity, factors influencing reproductive decisions may extend beyond individual couples. The result is that variation in fertility reflects both opportunities for market engagement and norms regarding resource pooling at the household level.

Furthermore, the tempo of economic and demographic change in mixed economies provides an opportunity to test competing predictions about the role of cultural evolution, as opposed to novel economic payoffs, in driving the spread and maintenance of fertility declines [14]. For example, a substantial decline in fertility may take place prior to women adopting low, parity-specific stopping targets, such as the two child norm [66]. In these cases, household reproductive decision-making may reflect economic trade-offs where households attempt to maximize livelihood diversity while minimizing the costs of too many offspring.

## Limitations

There are a number of limitations in our study. First, we model fertility, which is an individual-level behavior, at the household level. Aggregating fertility to the household-level collapses important between-individual variation that may shed light on how households allocate resources to offspring investment and end up with diverse economic portfolios. For example, longitudinal studies could identify how parental investment is divided within households and how that investment shapes later economic activities of adult members. This would also provide insight into how individuals actively strategize reproductive decisions around long-term household economic contexts. Second, we use income-per capita as a measure of household economic success. However, income may be an inadequate indicator of long-term economic stability, particularly in low and middle income countries.[67, 68]. Future research should link both fertility decisions and household economic diversity to more stable measures of long-term economic well-being, such as asset-based wealth or locally salient measures of economic success.

## Conclusion

Mixed economies present an environment of market opportunities and traditional economic production. Demographic characteristics of households can shape how households navigate trade-offs of engaging in different suites of economic activities. In these contexts, economic models of the fertility transition that focus on individual decision-making and wage-labor returns on investments in education may not capture household-level economic constraints or considerations. The results of this study demonstrate how fertility decisions can shape household composition that can directly and indirectly influence both livelihood diversity and income through a household. In this way, fertility becomes both an outcome and an input into economic and reproductive decisions-making.

## Supporting information

S1 File Anonymized data. (CSV)

S2 File (DOCX)
