# Supplementary material for: Balancing fertility and livelihood diversity in mixed economies
Source: PLoS One. 2021 Jun 24;16(6):e0253535. doi: 10.1371/journal.pone.0253535 (PMC8224957; doi:10.1371/journal.pone.0253535)
Supplement: S2 File — (DOCX) [file pone.0253535.s002.docx]

SUPLEMENTAL MATERIALS: BALANCING FERTILITY AND LIVELIHOOD DIVERSITY IN MIXED ECONOMIES

Joseph V. Hackman *

Karen L. Kramer

1. Sensitivity Analyses of Aggregating Household Fertility.
   1. Head of Household Fertility in 2010
   2. Average Completed Fertility (Women 40 yrs or older) in 2010
2. Including Proportion of Women in the Household

Sensitivity analyses of aggregating fertility

S1 Table and S2 Table show model results of sensitivity analyses where household fertility were aggregated in different ways. Focusing solely on head of household fertility in 2010 (S1 Table), results are consistent with those presented in the main text. Income is associated positively with livelihood diversity and negatively with household size. Additionally, household size is positively associated with livelihood diversity. Lastly, head of household fertility in 2010 was positively associated with household size in 2017. Differences in the stratified model were also consistent with the models from the main text. However, for newly established households, head of household fertility in 2010 was a significant predictor of household size in 2017.

| S1 Table – SEM Models with Head of Household Fertility in 2010 | | | | | | | | | | | | |
| --- | --- | --- | --- | --- | --- | --- | --- | --- | --- | --- | --- | --- |
|  |  | Full Model | | |  | Established Households | | |  | New Households | | |
| Coefficients |  | B | SE | P |  | B | SE | P |  | B | SE | P |
| **Income** |  |  |  |  |  |  |  |  |  |  |  |  |
| Intercept |  | 8.62 | 0.45 | 0.00 |  | 8.39 | 0.62 | 0.00 |  | 8.99 | 0.58 | 0.00 |
| Livelihood Diversity |  | 0.45 | 0.14 | 0.00 |  | 0.46 | 0.18 | 0.01 |  | 0.38 | 0.19 | 0.04 |
| Household Size 2017 |  | -0.16 | 0.06 | 0.01 |  | -0.13 | 0.09 | 0.17 |  | -0.17 | 0.07 | 0.01 |
| **Livelihood Diversity** |  |  |  |  |  |  |  |  |  |  |  |  |
| Intercept |  | 2.60 | 0.21 | 0.00 |  | 2.44 | 0.32 | 0.00 |  | 0.00 | 0.00 | 0.00 |
| Household Size 2017 |  | 0.18 | 0.04 | 0.00 |  | 0.20 | 0.06 | 0.00 |  | 0.16 | 0.06 | 0.01 |
| **Household Size in 2017** |  |  |  |  |  |  |  |  |  |  |  |  |
| Intercept |  | 2.21 | 2.46 | 0.37 |  | 2.44 | 0.32 | 0.00 |  | 2.75 | 0.25 | 0.00 |
| HH Fertility in 2010 |  | 0.44 | 0.10 | 0.00 |  | 0.42 | 0.10 | 0.00 |  | 0.54 | 0.24 | 0.03 |
| Age of HH |  | 0.00 | 0.12 | 1.00 |  | 0.17 | 0.16 | 0.30 |  | -0.15 | 0.41 | 0.71 |
| Age of HH^2 |  | -0.004 | 0.001 | 0.00 |  | -0.003 | 0.001 | .02 |  | .005 | 0.002 | 0.01 |
| Established HH |  | 1.99 | 0.56 | 0.00 |  |  |  |  |  |  |  |  |
| Model Fit Indices |  | Coef | P |  |  | Coef | P |  |  | Coef | P |  |
| Chi Square |  | 7.73  [ df=8] | 0.46 |  |  | 6.72 [ df=6] | 0.35 |  |  | 8.9  [ df=6] | 0.18 |  |
| RMSEA |  | 0.001 [90% CI=0.00-0.12] | 0.61 |  |  | 0.045 [90% CI=0.00-0.18] | 0.44 |  |  | 0.13 [90% CI=0.00-0.30] | 0.22 |  |
| R-squared |  |  |  |  |  |  |  |  |  |  |  |  |
| Income |  | 0.13 |  |  |  | 0.10 |  |  |  | 0.21 |  |  |
| Livelihod Diversity |  | 0.16 |  |  |  | 0.16 |  |  |  | 0.20 |  |  |
| Household Size |  | 0.32 |  |  |  | 0.33 |  |  |  | 0.24 |  |  |

Models that estimate household fertility using only women aged 40 or older in 2010 are again similar to those presented in the main text (S2 Table). For the full model, income, household size, and livelihood diversity show the same sign and magnitude of associations as those in the main text. Fertility in 2010 however, was not a statistically significant predictor of household size in 2017. However, fertility in 2010 is a significant predictor of household size in 2017 for established households.

| S2 Table -SEM Models with Average Completed Household Fertility in 2010 ( Woman>40 yrs old) | | | | | | | | | | | | | |
| --- | --- | --- | --- | --- | --- | --- | --- | --- | --- | --- | --- | --- | --- |
|  |  | Full Model (N=56) | | |  | Established Households (N=35) | | |  | New Households (N=21) | | | |
| Coefficients |  | B | SE | P |  | B | SE | P |  | B | SE | P |  |
| **Income** |  |  |  |  |  |  |  |  |  |  |  |  |  |
| Intercept |  | 8.99 | 0.31 | 0.00 |  | 8.94 | 0.35 | 0.00 |  | 9.04 | 0.63 | 0.00 |  |
| Livelihood Diversity |  | 0.39 | 0.10 | 0.00 |  | 0.37 | 0.11 | 0.00 |  | 0.40 | 0.21 | 0.06 |  |
| Household Size 2017 |  | -0.16 | 0.04 | 0.00 |  | -0.14 | 0.06 | 0.01 |  | -0.19 | 0.07 | 0.01 |  |
| **Livelihood Diversity** |  |  |  |  |  |  |  |  |  |  |  |  |  |
| Intercept |  | 2.36 | 0.26 | 0.00 |  | 1.99 | 0.42 | 0.00 |  | 0.00 | 0.00 | 0.00 |  |
| Household Size 2017 |  | 0.22 | 0.05 | 0.00 |  | 0.28 | 0.07 | 0.00 |  | 0.17 | 0.07 | 0.01 |  |
| **Household Size in 2017** |  |  |  |  |  |  |  |  |  |  |  |  |  |
| Intercept |  | 5.82 | 3.31 | 0.08 |  | 1.99 | 0.42 | 0.00 |  | 2.63 | 0.32 | 0.00 |  |
| HH Fertility in 2010 |  | 0.17 | 0.12 | 0.16 |  | 0.37 | 0.13 | 0.00 |  | -0.04 | 0.24 | 0.87 |  |
| Age of HH |  | -0.12 | 0.16 | 0.46 |  | 0.17 | 0.19 | 0.39 |  | -0.52 | 0.57 | 0.36 |  |
| Age of HH^2 |  | 0.00 | 0.00 | 0.93 |  | 0.00 | 0.00 | 0.00 |  | 0.00 | 0.00 | 0.00 |  |
| Established HH |  | 3.10 | 0.80 | 0.00 |  |  |  |  |  |  |  |  |  |
| Model Fit Indices |  | Coef | P |  |  | Coef | P |  |  | Coef | P |  |  |
| Chi Square |  | 5.643  [ df=8] | 0.69 |  |  | 8.97 [ df=6] | 0.18 |  |  | 7.63 [ df=6] | 0.27 |  |  |
| RMSEA |  | 0.001 [90% CI=0.00-0.12] | 0.77 |  |  | 0.119 [90% CI=0.00-0.27] | 0.22 |  |  | 0.114 [90% CI=0.00-0.32] | 0.30 |  |  |
| R-squared |  |  |  |  |  |  |  |  |  |  |  |  |  |
| Income |  | 0.25 |  |  |  | 0.25 |  |  |  | 0.25 |  |  |  |
| Livelihod Diversity |  | 0.26 |  |  |  | 0.29 |  |  |  | 0.24 |  |  |  |
| Household Size |  | 0.28 |  |  |  | 0.41 |  |  |  | 0.15 |  |  |  |

Including a measure of household composition did not qualitatively change the results. Proportion of women in the household did not have a significant effect on either income or livelihood diversity in any of the models.

| S3 Table - SEM Models including Proportion of Women in the Household | | | | | | | | | | | | |
| --- | --- | --- | --- | --- | --- | --- | --- | --- | --- | --- | --- | --- |
|  |  | Full Model (N=88) | | |  | Established Households (N=59) | | |  | New Households (N=29) | | |
| Coefficients |  | B | SE | P |  | B | SE | P |  | B | SE | P |
| **Income** |  |  |  |  |  |  |  |  |  |  |  |  |
| Intercept |  | 8.90 | 0.39 | 0.00 |  | 8.76 | 0.47 | 0.00 |  | 9.70 | 0.74 | 0.00 |
| Livelihood Diversity |  | 0.39 | 0.10 | 0.00 |  | 0.38 | 0.11 | 0.00 |  | 0.40 | 0.21 | 0.05 |
| Household Size 2017 |  | -0.16 | 0.04 | 0.00 |  | -0.14 | 0.06 | 0.02 |  | -0.25 | 0.08 | 0.00 |
| Proportion of Adults Female |  | 0.16 | 0.47 | 0.73 |  | 0.28 | 0.52 | 0.59 |  | -1.13 | 1.01 | 0.26 |
| **Livelihood Diversity** |  |  |  |  |  |  |  |  |  |  |  |  |
| Intercept |  | 2.52 | 0.39 | 0.00 |  | 2.21 | 0.60 | 0.00 |  | 2.52 | 0.56 | 0.00 |
| Household Size 2017 |  | 0.21 | 0.05 | 0.00 |  | 0.37 | 0.13 | 0.00 |  | 0.18 | 0.07 | 0.01 |
| Proportion of Adults Female |  | -0.28 | 0.61 | 0.65 |  | 0.17 | 0.19 | 0.39 |  | 0.20 | 1.06 | 0.85 |
| **Household Size in 2017** |  |  |  |  |  |  |  |  |  |  |  |  |
| Intercept |  | 5.82 | 3.31 | 0.08 |  | 1.03 | 4.68 | 0.83 |  | 14.06 | 8.75 | 0.11 |
| HH Fertility in 2010 |  | 0.17 | 0.12 | 0.16 |  | 0.37 | 0.13 | 0.00 |  | -0.04 | 0.24 | 0.87 |
| Age of HH |  | -0.12 | 0.16 | 0.46 |  | 0.17 | 0.19 | 0.39 |  | -0.52 | 0.57 | 0.36 |
| Age of HH^2 |  | 0.00 | 0.00 | 0.93 |  | 0.00 | 0.00 | 0.17 |  | 0.01 | 0.01 | 0.51 |
| Established HH |  | 3.10 | 0.80 | 0.00 |  |  |  |  |  |  |  |  |
| Model Fit Indices |  | Coef | P |  |  | Coef | P |  |  | Coef | P |  |
| Chi Square |  | 15.36 (9) | 0.08 |  |  | 9.39 (7) | 0.23 |  |  | 29.53 (7) | 0.00 |  |
| RMSEA |  | 0.08 [90% CI=0.00-0.19] | 0.28 |  |  | 0.10 [90% CI=0.00-0.24] | 0.28 |  |  | 0.11 [90% CI=0.00-0.32] | 0.30 |  |
| R-squared |  |  |  |  |  |  |  |  |  |  |  |  |
| Income |  | 0.25 |  |  |  | 0.25 |  |  |  | 0.25 |  |  |
| Livelihod Diversity |  | 0.31 |  |  |  | 0.29 |  |  |  | 0.24 |  |  |
| Household Size |  | 0.28 |  |  |  | 0.41 |  |  |  | 0.15 |  |  |
